# Annexin A1 and the regulation of innate and adaptive immunity

**DOI:** 10.3389/fimmu.2012.00354

**Published:** 2012-11-27

**Authors:** Felicity N. E. Gavins, Michael J. Hickey

**Affiliations:** ^1^Centre for Neuroinflammation and Neurodegeneration, Division of Brain Sciences, Imperial College LondonLondon, UK; ^2^Centre for Inflammatory Diseases, Monash University Department of Medicine, Monash Medical CentreMelbourne, VIC, Australia

**Keywords:** annexin A1, inflammation, innate immunity, adaptive immunity, formyl peptide receptor

## Abstract

Inflammation is the body’s way of defending itself against noxious stimuli and pathogens. Under normal circumstances, the body is able to eliminate the insult and subsequently promote the resolution of inflammation and the repair of damaged tissues. The concept of homeostasis is one that not only requires a fine balance between both pro-inflammatory mediators and pro-resolving/anti-inflammatory mediators, but also that this balance occurs in a time and space-specific manner. This review examines annexin A1, an anti-inflammatory protein that, when used as an exogenous therapeutic, has been shown to be very effective in limiting inflammation in a diverse range of experimental models, including myocardial ischemia/reperfusion injury, arthritis, stroke, multiple sclerosis, and sepsis. Notably, this glucocorticoid-inducible protein, along with another anti-inflammatory mediator, lipoxin A_4_, is starting to help explain and shape our understanding of the resolution phase of inflammation. In so doing, these molecules are carving the way for innovative drug discovery, based on the stimulation of endogenous pro-resolving pathways.

## INTRODUCTION

Inflammation is essential for the body to maintain homeostasis and recover from tissue injury or noxious stimuli. However, a key step in the inflammatory response is the resolution phase. Overly aggressive or prolonged inflammation, which fails to resolve, can lead to tissue destruction (Serhan and Chiang, 2008). The resolution of inflammation was once thought to be a passive process, but is now deemed to be very much an active phenomenon, and one that is tightly controlled by endogenous “pro-resolving” mediators. Resolution is accepted as one of the four major outcomes for acute inflammation, the others being progression to chronic inflammation, scarring, and fibrosis (Cotran et al., 1999; Kohli and Levy, 2009).

Over the years, interest has focused on anti-inflammatory mediators that have pro-resolving properties. In particular, endogenous anti-inflammatory molecules, such as the glucocorticoid-regulated protein annexin A1 (AnxA1), have particular appeal for drug discovery programs, based on the contention that drugs founded on endogenous anti-inflammatory molecular pathways could mimic their pro-resolution effects while potentially having fewer side effects than existing therapeutic agents.

This review will focus on AnxA1, its history, mechanism of action and its role in both innate and adaptive immunity and how this 37 kDa protein has potential in drug discovery.

## THE HISTORY OF ANNEXIN A1

In the late 1970s, a new protein was discovered and characterized by its ability to quash eicosanoid generation by affecting phospholipase A_2_ (PLA_2_) activity. The action on arachidonate and eicosanoid release *in vitro*, e.g., the inhibition of PGE_2_ and LTB_4_ release by monocytes and neutrophils (Parente et al., 1984) was accompanied by an inhibitory effect in experimental models of inflammation *in vivo* (e.g., TXA_4_ release from perfused guinea pig lungs; Cirino et al., 1987). Different names were proposed for this new protein, the molecular weight of which ranged from 15 to 40 kDa: macrocortin (because it was isolated from peritoneal exudates from glucocorticoid-treated rats), renocortin (released rat renal medulla cells; Russo-Marie and Duval, 1982), or lipomodulin (released from isolated neutrophils; Hirata, 1981), but it was decided that it should be termed lipocortin 1. It was accepted that these three proteins were functionally identical and all active fragments of the same precursor, thus it was agreed that a uniform name should be chosen: lipocortin (Di Rosa et al., 1984). In 1986, lipocortin was cloned (Wallner et al., 1986) and the sequence was termed lipocortin 1. Today, the name AnxA1 has been agreed on as a more appropriate choice, due to the ability of this protein to “annex” phospholipid membranes. Following on from this, AnxA1 has been shown to mimic the anti-inflammatory effect of glucocorticoids in number of *in vitro* and *in vivo* studies (the gene encoding AnxA1 is located on chromosome 19q24).

Annexin A1 is a 37-kDa member of the annexin superfamily of calcium- and phospholipid-binding proteins, of which there are currently 13 members. It consists of 346 amino acids and is made of four repeated sequences, which are arranged around a core [which represents the large majority (≥80%) of the protein], giving the protein a “doughnut” shape. At low Ca^2+^ concentrations, the N-terminal domain is embedded within the pore, but elevations in [Ca^2+^] (≥1 mM, e.g., as in plasma or other biological fluids) expose this region and may thereby influence the biological activity of the protein (Rosengarth et al., 2001a,b). All members of the annexin superfamily comprise of a core domain made up of four similar repeats (six repeats in the case of AnxA6), each approximately 70 amino acids long. The N-terminal region of each member of the annexin superfamily is unique and as such represents its own fingerprint, and confers biological activity. It is, however, still unknown as to whether there is any close regulation among members of the annexin family. It is likely that is the case, e.g., in the case of AnxA2, cells appear to require it as a structural/scaffolding protein that stabilizes and/or regulates the dynamics of certain membrane domains. Thus, it is probable that AnxA2 shares its activity with other annexins (Rescher and Gerke, 2004).

## BIOLOGY OF AnxA1

Within peripheral blood cells, under resting conditions, AnxA1 is mainly expressed in subcellular granules of neutrophils, eosinophils, and monocytes, with small amounts expressed in specific subsets of lymphocytes (Goulding et al., 1990; Morand et al., 1995; Rescher and Gerke, 2004; Spurr et al., 2011). T cells and mast cells express the protein, although B cells express it at low levels, and platelets do not (Cirino et al., 1987; Morand et al., 1995; Oliani et al., 2000; Rescher and Gerke, 2004). Cell differentiation (such as monocytes maturing into macrophages) tends to be associated with higher levels of expression of AnxA1, as demonstrated in studies showing that levels of AnxA1 expression are lower in monocytes relative to those in macrophages from the same donor (Perretti and Flower, 1996).

AnxA1 is undetectable in plasma, but is found in many tissues, including the lung, bone marrow, and intestine, at concentrations <50 ng/ml, with the highest levels in seminal fluid (150 µg/ml).

## AnxA1 AND THE INNATE IMMUNE RESPONSE

### THE ROLE OF AnxA1

Since its discovery, AnxA1 has been shown to be capable of modulating a number of biological events, including both acute (Gavins et al., 2003) and chronic (Goulding et al., 1998) inflammation, ischemia/reperfusion injury (D’Amico et al., 2000; La et al., 2001; Gavins et al., 2003), pain (Marchand et al., 2005), fever (Lim and Pervaiz, 2007), intracellular vesicle trafficking (Gerke et al., 2005), arachidonic acid release (Croxtall et al., 2000), leukocyte migration (Williams et al., 2010), and tissue growth and apoptosis (Petrella et al., 2005; Scannell and Maderna, 2006). AnxA1 may also play a role in the regeneration of skeletal muscle tissue by stimulating the migration of satellite cells via the modulation of myoblast cell differentiation, which in turn causes skeletal muscle differentiation (Hawke and Garry, 2001; Bizzarro et al., 2012). In addition, AnxA1 has a valuable role in inhibiting the negative feedback effects of glucocorticoids on the release of corticotrophin (ACTH) and hypothalamic-releasing hormones (Buckingham et al., 2006), and also affects a number of mediators that are involved in the inflammatory response, including cyclo-oxygenase-2 (Cox-2) and inducible nitric oxide synthase (iNOS; Minghetti et al., 1999; Ferlazzo et al., 2003; Perretti and Dalli, 2009).

Evidence indicates that AnxA1 may also play an important role in tumor development and progression, with AnxA1 levels being up- and down-regulated in different cancers, e.g., the loss of AnxA1 expression in prostate cancer correlates with an early onset of tumorigenesis (Xin et al., 2003). The fact that this protein also contains phosphorylation sites that can be phosphorylated by a number of proliferative signaling molecules, including PKC and EGF receptor tyrosine kinase, suggests that AnxA1 may also have a role in signaling pathways that are important in cancer (Alldridge et al., 1999; Hsiang et al., 2006). However, further work is required to define the actions of AnxA1 in this setting.

### AnxA1 AND CELL RECRUITMENT AND MIGRATION

**FIGURE 1:**
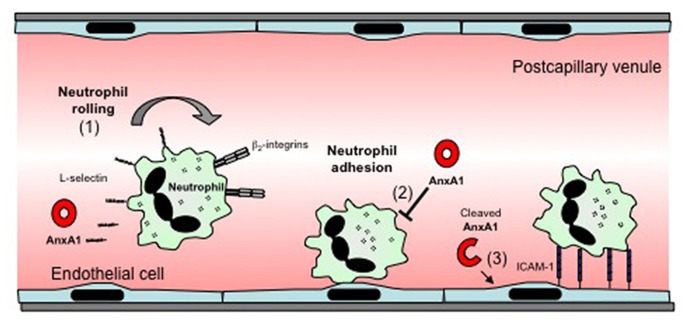
**Effects of AnxA1 on leukocyte–endothelial cell interactions**. The effects of AnxA1 on leukocyte endothelial cell interactions include: (1) induction of L-selectin-shedding, which may reduce leukocyte rolling. (2) Inhibition of leukocyte adhesion or detachment of already adherent leukocytes, an effect associated with reduced transmigration. (3) A novel cleaved form of AnxA1 has been reported to act on endothelial cells to promote ICAM-1 clustering around transmigrating neutrophils, facilitating transmigration.

AnxA1 has been shown to induce a number of effects relating to the adhesion and migration of leukocytes, processes that represent fundamental steps in the development of the inflammatory response. These include induction of L-selectin shedding by neutrophils, and detachment of adherent leukocytes from the endothelium (**Figure 1**). These actions contribute to the ability of AnxA1 to restrict leukocyte transmigration and recruitment during inflammation. AnxA1 has also been shown to reduce α4β1 integrin-dependent monocyte adhesion and migration (Cote et al., 2010). This anti-migratory capacity extends to other cell types such as endothelial cells (Cote et al., 2010). However, in contrast to its predominantly inhibitory effects on these processes, a recent study demonstrated that a novel cleavage product from the C terminus of AnxA1, released by activated neutrophils, acts to promote neutrophil transmigration by promoting clustering of intracellular adhesion molecule-1 (ICAM-1) on the endothelial cell surface around migrating neutrophils (Williams et al., 2010).

### EXTERNALIZATION OF AnxA1

In order for AnxA1 to exert its anti-inflammatory effects, it must be externalized by its cellular sources. However, AnxA1 lacks a signal peptide (similar to other proteins such as interleukin-1; Muesch et al., 1990; Christmas et al., 1991), and as such, cannot be exported through the classical secretory pathway, but rather by exocytosis. Upon cellular activation, AnxA1 is released from its storage site and translocates to the membrane, where it is secreted via different pathways, depending upon the cell type involved. In macrophages, the ATP-binding cassette (ABC) transporter system is responsible for the secretion of AnxA1 (Wein et al., 2004). In the neutrophil, AnxA1 is stored in gelatinase granules, and upon neutrophil-activating events, such as adhesion to the endothelial cell surface, it is rapidly mobilized to the outside leaflet of the plasma membrane (Perretti et al., 1996). In this location, the intact 37 kDa AnxA1 undergoes conformational change (Rosengarth et al., 2001a,b), exposing the N-terminal region, resulting in a structure which evidence indicates is the active form of AnxA1. In addition AnxA1 can be cleaved into a 33-kDa fragment. Several enzymes have been suggested to cause the cleavage, including elastase (Rescher et al., 2006), a metalloprotease (proposed to cleave the first seven amino acids of the AnxA1 terminus; Movitz et al., 1999), and proteinase 3 (Vong et al., 2008). It is still unknown whether the cleavage process occurs (1) to allow AnxA1 to act as a pro-drug via the production of a bioactive fragment, or (2) to produce homeostasis by limiting the action of AnxA1 (Perretti and Gavins, 2003; Pederzoli-Ribeil et al., 2010). Supporting the latter concept, a modified recombinant form of AnxA1 resistant to proteinase 3-mediated cleavage has been shown to have longer-lasting effects on neutrophil adhesion *in vivo*, relative to native AnxA1 (Pederzoli-Ribeil et al., 2010). In addition, of interest, in the case of the neutrophil, it has recently been shown that AnxA1 externalization can occur without interaction with an endothelial monolayer indicating that cellular adhesion to the endothelium is not required for release of AnxA1 (Vong et al., 2008).

Microparticle release may be an additional alternative mode of AnxA1 release from neutrophils. Microparticles are small vesicles released from activated cells, and neutrophil-derived microparticles have been shown to be rich in AnxA1 (Dalli et al., 2008). Furthermore, neutrophil-derived microparticles have been shown to inhibit neutrophil–endothelial cell interactions under flow *in vitro*, an effect dependent on AnxA1 present in the microparticles. These observations indicate that AnxA1-containing microparticles may be a critical source of functionally-relevant AnxA1 produced by neutrophils.

### MECHANISMS OF ACTION OF AnxA1

Until 2000, the way in which AnxA1 mediated its cellular effects remained unclear. However, a seminal paper by Gerke and colleagues demonstrated that formyl peptide receptor (FPR) antagonists [butyloxycarbonyl (Boc) derivatives] blocked the anti-migratory effects of both intact AnxA1, and the AnxA1-derived peptide Ac2-26, on human neutrophils, as well as modulating the effects of AnxA1 on calcium flux and L-selectin shedding (Walther et al., 2000). These *in vitro* effects were mirrored *in vivo* using Fpr1^–/–^ mice, which displayed an attenuation of the inhibitory actions of AnxA1 and peptide Ac2-26 in a model of peritonitis (Perretti et al., 2001). Together, these studies were the first to demonstrate a role for FPRs in mediating the cellular effects of AnxA1.

### THE FORMYL PEPTIDE RECEPTORS

**FIGURE 2:**
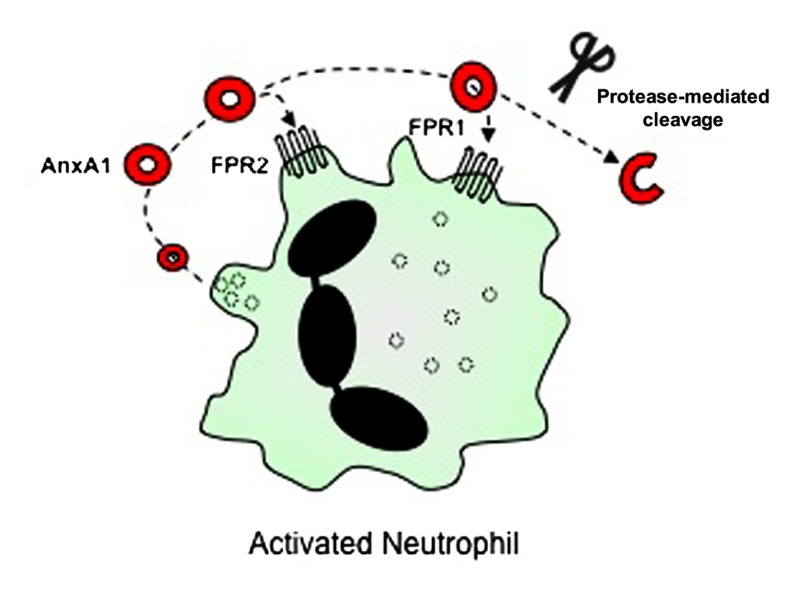
** Cellular receptors for AnxA1**. AnxA1 is released from granules of activated neutrophils into the extracellular space, where it is then able to bind to G protein-coupled receptors including FPR2/ALXR and FPR1, thereby mediating cellular effects via autocrine (and paracrine) pathways. In addition, proteases released from activated neutrophils can cleave full-length AnxA1, although the purpose of this process (for AnxA1 to act as a pro-drug, or whether it is a catabolic event terminating its action) is still under debate.

Evidence now indicates that AnxA1 mediates most of its cellular effects via interaction with FPRs (**Figure 2**). The FPRs are a family of seven transmembrane domain, G protein-coupled receptors, that are expressed mainly in mammalian phagocytic leukocytes, where they serve to induce responses to various ligands, including the bacteria-derived peptide, fMLF (Ye et al., 2009). Three FPR receptors exist in the human, namely FPR1, FPR2/ALXR (which shares 69% amino acid sequence homology with FPR1, and is also known as the LXA_4_ receptor) and FPR3 (which shares 56% amino acid similarity to FPR1 and 72% to FPR2/ALXR; Brink, 2003; Ye et al., 2009; Gavins, 2010). The receptor story in the mouse is rather more complicated with the gene cluster having undergone differential expansion. It is now agreed that *Fpr1*, the murine ortholog of human *FPR1*, is 77% identical to human *FPR1* (Brink, 2003; Ye et al., 2009; Gavins, 2010) and *Fpr3* is 73% identical to human *FPR2/ALXR*. Fpr2 binds fMLF with low affinity (Brink, 2003; Ye et al., 2009; Gavins, 2010). *Fprs3*,* 4*, *6*, and *7* appear not to have direct counterparts in the human genome (Yang et al., 2004; for further review of these receptors, see Brink, 2003; Ye et al., 2009; Gavins, 2010).

The FPRs are primarily coupled through pertussis toxin-insensitive G proteins (G_*I*_α_2_, G_*I*_α_3_) to the activation of phospholipase C and ultimately release Ca^2+^ from intracellular stores (Wenzel-Seifert et al., 1999). This release of Ca^2+^ causes the opening of the store-operated Ca^2+^ channel in the plasma membrane allowing further Ca^2+^ influx into the cell (Ye et al., 2009). Other intracellular signaling effects of ligand binding to FPRs include tyrosine kinase-mediated phosphorylation of PLA, PLD, and members of the MAP kinase family (Brink, 2003; Ye et al., 2009; Gavins, 2010), and signaling through Cdc42 to activate Rac- and ARP2/3-dependent pathways leading to actin nucleation (VanCompernolle et al., 2003).

### FPRs AND AnxA1

Upon binding to the FPRs on neutrophils, AnxA1 induces responses such as L-selectin shedding and detachment from the endothelium (Perretti et al., 1996; Lim et al., 1998; Gavins et al., 2003). It has also been shown to cause desensitization of the receptor toward the fMLF stimulus (D’Acquisto et al., 2008). Evidence indicates that much of the AnxA1 that mediates this effect comes from the leukocyte itself (D’Acquisto et al., 2008). This autocrine/paracrine effect of AnxA1, which inhibits the process of leukocyte transmigration, has been suggested specifically for leukocytes (Maderna et al., 2005), but it is likely that it occurs in a number of different cells, including macrophages (Babbin et al., 2006) and epithelial cells (Solito et al., 2000).

The specific member(s) of the FPR family mediating the effect of AnxA1 and its mimetic peptides appear to be not only tissue-specific, but also different depending upon whether full-length AnxA1 or AnxA1-derived peptide is used (Yang et al., 2004; Gavins et al., 2005a,b). For example, in a model of peritoneal inflammation induced by zymosan A, the anti-migratory effect of peptide Ac2-26 was absent in Fpr1 null mice, whereas the response to whole protein was not (Perretti et al., 2002). In the murine mesentery, both Fpr1 and Fpr2 appear to be involved, with the latter receptor being more functionally involved in the detachment of leukocytes from the endothelium (Gavins et al., 2003).

Further evidence of an interaction between AnxA1 and FPRs came from immunoprecipitation studies in which neutrophil-derived AnxA1 could be immunoprecipitated with FPR2/ALXR when the leukocytes were adherent to endothelial monolayers (Solito et al., 2003). These data were supported by ligand-binding studies using transfected cells stably expressing members of the FPR family.

With respect to formylated mitochondria proteins, studies have reported that mitochondrial-derived FPR1 ligands function as chemotactic damage-associated molecular pattern molecules (DAMPs, also known as or alarmins or danger signals; McDonald et al., 2010; Reddy and Standiford, 2010). DAMPs have pro-inflammatory activity and are released or generated after injury, thus activating the innate immune system (Chen and Nunez, 2010; Rock et al., 2010). To our knowledge, no studies have shown an involvement of AnxA1 in modulating the inflammatory response to injury associated with formylated mitochondria proteins.

It is worth pointing out that LXA_4_ and its analogs have opposing effects to AnxA1 and its mimetic peptides, despite both interacting with FPR2/ALXR on peripheral blood leukocytes. Whereas AnxA1 and its peptides (Walther et al., 2000; Jozsef et al., 2002; Solito et al., 2003) cause L-selectin shedding by both neutrophils and monocytes *in vitro*, LXA_4_ and its analogs increase basal cell surface levels of L-selectin (Gavins et al., 2005b). These observations may be due to ligand-specific conformation that may occur with the same receptor, resulting in ligand-specific signal transduction responses that yield specific cellular effects unique to each particular ligand (Gavins et al., 2005a).

### AnxA1^–/–^ MOUSE

The development of the AnxA1^–/–^ mouse (generated by homologous recombination, with a transgenic gene that disrupted the endogenous AnxA1 gene, and a LacZ gene under the control of the AnxA1 promoter; Hannon et al., 2003) has led to a greater understanding of the roles played by AnxA1 in inflammation, and has demonstrated that, in general, where AnxA1 is absent, inflammation is exacerbated and prolonged. This has important significance in exploiting the biological properties of AnxA1 in development of novel anti-inflammatory agents. AnxA1^–/–^ mice have a heightened inflammatory response as displayed by increased leukocyte transmigration (Chatterjee et al., 2005), higher levels of inflammatory markers in a model of localized joint inflammation (Reddy and Standiford, 2010), increased neurological deficit in a stroke model (Gavins et al., 2007) and delayed repair in a model of colitis (Babbin et al., 2008). *In vitro*, neutrophils from these mice have a greater propensity for chemotaxis and higher CD11b expression. In addition, consistent with these observations, absence of AnxA1 also leads to increased inflammation and in some cases a higher mortality in life-threatening inflammation-associated conditions, e.g., stroke (Reddy and Standiford, 2010). In many of these inflammatory conditions and situations, the administration of exogenous AnxA1 is able to rescue the phenotype in AnxA1^–/–^ mice (Gavins et al., 2003).

### PRO-INFLAMMATORY ACTIONS OF AnxA1

In contrast to much of the research on AnxA1 and innate immunity, some studies have pointed to the capacity of this protein and its cleavage products to mediate pro-inflammatory actions. For example, as described earlier, Williams et al. (2010) found that that a novel cleavage product of AnxA1 promotes neutrophil transmigration via effects on endothelial ICAM-1. Similarly, a peptide from the N-terminal domain of AnxA1 has been found to promote leukocyte chemotaxis via FPR family members (Ernst et al., 2004). AnxA1 has also been found to be released from rheumatoid arthritis synovial fibroblasts (RASF) following TNF-mediated activation, and to promote RASF matrix metalloproteinase-1 secretion (Tagoe et al., 2008). Furthermore, the absence of AnxA1 has recently been reported to be without effect in a T cell-independent, antibody transfer model of arthritis, indicating that under some conditions, the role of AnxA1 is minimal (Patel et al., 2012). These studies demonstrate the multifaceted nature of the actions of this intriguing molecule and its cleavage products, and highlight the necessity for detailed studies in the complex *in vivo* environment in order to fully understand their actions in inflammatory responses.

## AnxA1 AND THE ADAPTIVE IMMUNE RESPONSE

As already described, much of the research on AnxA1 has focused on its effects in forms of inflammation mediated by neutrophils and monocyte/macrophages. However, a growing body of evidence indicates that AnxA1 also modulates the adaptive immune response and tissue injury in models of inflammation induced by activation of the adaptive immune system (D’Acquisto et al., 2008). A notable difference between these sets of observations is that studies of innate responses routinely report that AnxA1 mediates anti-inflammatory effects, while data emerging from studies on the role of AnxA1 in the adaptive immune response have been much less consistent. In this section, we will examine these studies and summarize the evidence regarding the actions of AnxA1 in the adaptive response.

### POSITIONING AnxA1 IN THE DEVELOPMENT OF THE ADAPTIVE RESPONSE

To understand the potential actions of AnxA1 in adaptive immunity, i.e., responses mediated via antigen recognition by T cells, B cells, and antibody, it is important to understand the typical steps in the development of an adaptive immune response. After emerging from the thymus, naïve T cells migrate to peripheral lymphoid organs, where they can undergo activation upon exposure to cognate antigen presented via dendritic cells. Similarly, immature B cells migrate from the bone marrow into the periphery and subsequently undergo antigen-dependent activation promoting their maturation into antibody-secreting cells. This initial phase is termed the activation or sensitization phase. As a result of these activation processes, the immune response is primed to react rapidly to re-exposure to the same antigen. This antigen-specific “effector response” commonly occurs in peripheral tissues in response to local re-application of the same cognate antigen, resulting in a long-lived inflammatory response at the site of exposure. Examples of experimental models used to investigate the mechanisms of this process include experimental autoimmune encephalomyelitis (EAE), antigen-induced arthritis, and dermal contact hypersensitivity, in which the effector phases target the brain, joint, and skin, respectively (Liu et al., 1998; Reddy and Standiford, 2010; Deane et al., 2012). The complexity of these multi-step processes allows numerous opportunities for AnxA1 to exert effects.

It is reasonable to assume that for AnxA1 to participate in development of the adaptive response, that the key cellular players would express AnxA1 and/or FPR2/ALXR. As such, it was recognized many years ago that AnxA1 is expressed constitutively by T cells, although at ~25% of the levels expressed in neutrophils (Goulding et al., 1990; Morand et al., 1995; Perretti and Flower, 1996; Paschalidis et al., 2009; Spurr et al., 2011). In addition, while unstimulated T cells have been shown to express FPR2/ALXR at low or negligible levels, following stimulation they increase surface expression of FPR2/ALXR within 30 min, maintaining elevated expression for several hours (D’Acquisto et al., 2007a). In addition, T cells release AnxA1 following activation of the T cell receptor (TCR; D’Acquisto et al., 2007b). Recent studies have performed more detailed analyses of the expression of AnxA1, and its receptor, in a range of T cell subsets. This work reveals that CD4^+^ T cells express slightly more AnxA1 than CD8^+^ T cells, predominantly intracellularly. Further analysis of the CD4^+^ subsets demonstrated that activated and memory cells express more AnxA1 than naïve cells, both intracellularly and on the cell surface. FPR2/ALXR is also expressed at a higher level in post-activation T cells, although the scale of increase is smaller relative to that of AnxA1 (Spurr et al., 2011). B cells also express intermediate levels of AnxA1 but low levels of FPR2/ALXR in the absence of stimulation (Spurr et al., 2011). Dendritic cells have also been shown to constitutively express and release AnxA1 (Huggins et al., 2009). Together these findings raise the possibility that AnxA1 may have important actions on these cell types.

### AnxA1 AND SUPPRESSION OF THE ADAPTIVE RESPONSE

**FIGURE 3:**
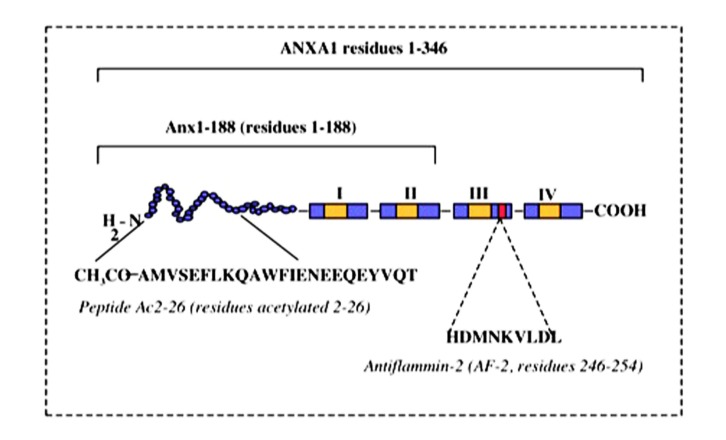
** Peptide structure of AnxA1**. Schematic representation of AnxA1 and peptides derived from the primary sequence.

Early studies of the actions of AnxA1 (“lipomodulin”) provided evidence that AnxA1 has the capacity to promote the development of anti-inflammatory regulatory T cells. This effect, detected using thymocyte-based suppression assays, was complex in that it occurred under moderate stimulatory conditions, but was reversed in response to strong T cell stimulation via high concentration Con A (Hirata and Iwata, 1983). Nevertheless, this study was interpreted to indicate that at least under some activating conditions, AnxaA1 promotes the generation and/or maturation of “suppressor” T cells. These findings were consistent with AnxA1 acting to limit T cell-dependent responses under some conditions. Parallel findings were reported by Gold et al. (1996), who observed suppression of T cell proliferation in response to exogenous AnxA1, using antigen-stimulated rat T cell lines. Similarly, proliferation and activation of peripheral blood mononuclear cells (PBMC) from atopic individuals were found to be inhibited in the presence of exogenous AnxA1-derived peptides, Ac2-26 and antiflammin-2 (AF-2; aa246–254; **Figure 3**; Kamal et al., 2001). The use of PBMC in this study made it unclear which leukocyte subsets were involved in the response. However, these cells were stimulated with peptide antigens known to induce T cell responses in the donors (house dust mite allergen – Der p; purified protein derivative of *Mycobacterium tuberculosis* – PPD), ensuring that stimulation occurred in an antigen-specific, T cell-dependent manner. While this did not exclude actions of AnxA1 in other leukocyte subsets present in the PBMC preparation, it ensured that the T cell was the primary target of the activation.

This work was extended into the *in vivo* setting by Yang et al., who examined AnxA1^–/–^ mice in an antigen-induced model of arthritis mediated by initial sensitization to mBSA, and subsequent local intra-articular mBSA challenge (Reddy and Standiford, 2010). In this study, absence of AnxA1 was associated with increased joint inflammation, consistent with AnxA1 acting to limit this adaptive response. Interestingly, in parallel with this result, antigen-specific IgG levels were reduced in AnxA1^–/–^ mice, despite the exacerbation of inflammation. This finding raises the intriguing possibility that under some circumstances, AnxA1 has the opposing effect on B cell function and generation of humoral immunity as it does on T cell-dependent inflammation. More recently, the role of AnxA1 was examined in an ovalbumin (OVA)-induced model of airways hyperresponsiveness (Ng et al., 2011). Similar to the arthritis study, the absence of AnxA1 was associated with evidence of increased allergic inflammation in the OVA-challenged lung, including increased eosinophil recruitment, IL-4 production, and airways dysfunction. However, this study revealed unanticipated complexities in the actions of AnxA1. Firstly, despite the exacerbation of inflammation in AnxA1^–/–^ mice, antigen-induced activation of MAP kinase and NFκB pathways in lungs of these animals were markedly blunted relative to that in wild-type mice. Furthermore, the absence of AnxA1 was associated with exacerbated airway reactivity in naïve mice, i.e., mice those had not undergone sensitization to antigen. These findings pointed to unidentified actions of AnxA1 in pulmonary physiology, presumably unrelated to its actions in the immune system.

Together these studies provide evidence that AnxA1 acts to limit inflammation in models associated with activation of the adaptive immune system. However, the molecular mechanisms of these effects, and the range of cellular targets of AnxA1, remain to be fully characterized. Furthermore, as these experiments predominantly focused on whole animal approaches, they did not determine whether AnxA1 mediated these effects directly in T cells or other cells involved in development of the adaptive response. Given the broad range of actions of AnxA1, it is conceivable that the absence of AnxA1 from cells in the target tissue with key roles in the effector response, potentially including endothelial cells or other effector leukocyte populations such as neutrophils, may have contributed to the exacerbation of inflammation in the absence of AnxA1. This is particularly important in the context of an unexpected association between exacerbated inflammation and defective T cell activation.

### AnxA1 AND ACTIVATION OF THE Th1 RESPONSE

In contrast to the work described above, a further group of studies has provided evidence that AnxA1 promotes T cell activation and T cell-dependent inflammation. In initial studies, D’Acquisto et al. (2007b) examined the effects of recombinant AnxA1 on T cells undergoing anti-CD3/CD28 activation, observing increased proliferation and IL-2 production in response to AnxA1. The effect of AnxA1 was most evident in T cells undergoing sub-maximal stimulation, while AnxA1 alone was insufficient to activate T cells. This effect was associated with increased TCR-dependent signaling, as demonstrated by activation of AP-1, NFκB, and NFAT. T cell activation also rapidly increased surface expression of FPR2/ALXR and exteriorization and release of AnxA1, and increased phosphorylation of ERK and Akt, signaling pathways downstream of FPR2/ALXR.

Numerous studies have demonstrated that following activation, CD4^+^ T cells differentiate down distinct lineages (Th1, Th2, Th17) defined by the profile of the cytokines they produce. Moreover, *in vivo* the composition of these subsets determines the phenotype of the resultant effector response (Iwakura et al., 2008; Zhu and Paul, 2010). D’Acquisto et al. (2007b) investigated the effect of AnxA1 on T cell differentiation and observed that exogenous AnxA1 favored generation of the IFNγ-producing Th1 subset, while inhibiting development of the IL-4-producing Th2 subset. To test if this subset “skewing” also occurred *in vivo*, they examined the Th1-dominant collagen-induced model of arthritis (CIA) and observed that exogenously administered AnxA1 induced exacerbation of joint inflammation associated with increased lymphocyte production of the key Th1 cytokine, IFN-γ. In this experiment, recombinant AnxA1 was administered twice daily commencing immediately after immunization. The presence of exogenous AnxA1 at these early stages of immunization in parallel with the significant enhancement of disease severity suggests that T cell differentiation was an important target of AnxA1 in this model. These findings support the hypothesis that AnxA1 alone does not directly activate T cells, but enhances activation and Th1 differentiation in the context of conventional T cell activation.

In complementary studies, the same group examined responses of T cells from AnxA1^–/–^ mice (D’Acquisto et al., 2007a). Notably, AnxA1^–/–^ T cells were found to undergo a significantly higher rate of basal proliferation relative to wild-type T cells. However, following non-specific activation, AnxA1^–/–^ T cells displayed significant reductions in proliferation and cytokine production relative to comparably-activated wild-type cells (D’Acquisto et al., 2007b). These findings indicate that the actions of AnxA1 differ according to the state of T cell activation. Consistent with previous work, in activated cells the absence of AnxA1 was associated with reduced activation of signaling pathways downstream of the TCR and Fpr2/ALXR (AP-1, NFκB, NFAT, MAP kinase, Akt; Ng et al., 2011). Mirroring the effect of exogenous AnxA1 on Th1 development, the absence of AnxA1 favored development of IL-4/IL-13-generating Th2 cells (D’Acquisto et al., 2007b). In addition, AnxA1^–/–^ T cells produced less IL-17, suggesting that AnxA1 also supports development of the Th17 CD4^+^ T cell phenotype. Data from a Th2-dependent model of allergic peritonitis supported this idea in that AnxA1^–/–^ mice showed increased effector phase leukocyte recruitment, most prominently of eosinophils. These studies provide evidence that the actions of AnxA1 on T cell activation are subset-specific, promoting development of the Th1 and Th17 subsets, while inhibiting development of the Th2 response.

This work was extended to the examination of T cell-mediated inflammation of the central nervous system (Paschalidis et al., 2009). Using the myelin oligodendrocyte glycoprotein (MOG)-induced model of EAE, Paschalidis et al. (2009) observed a mild protection from clinical disease in AnxA1^–/–^ mice, most prominently in the later phase of the disease. This was associated with reduced antigen-specific recall proliferation and IL-2 production, and reduced T cell production of Th1 and Th17 cytokines in AnxA1^–/–^ mice.

Together this body of work indicates that endogenous AnxA1 acts to restrict Th2 development, favoring development of Th1- and Th17-mediated responses. These findings raise the possibility of AnxA1 being a therapeutic target in autoimmune diseases characterized by inappropriate activity of Th1/Th17 subsets.

### AnxA1 AND THE ADAPTIVE RESPONSE – WHERE TO FROM HERE?

Taken together, some inconsistencies between these studies remain, indicating that further work is required to clarify the actions of AnxA1 in T cell-mediated responses. One of the fundamental questions that emerges is, via what mechanism does AnxA1 promote Th1/Th17 development while inhibiting Th2 development? To this end, there is a growing body of evidence that control of T cell activation via modulation of T cell signaling is a key component of this action of AnxA1. In T cells exposed to a range of stimuli, AnxA1 has been found to modulate activation of Akt and ERK MAP kinase, pathways that are central to the TCR-mediated response (D’Acquisto et al., 2007a,b; Paschalidis et al., 2009). Further work is required to determine how these intracellular effects control the polarization of CD4^+^ T cells during development of the immune response. An additional aspect that remains unclear is what are the actions of AnxA1 in different cell types? In none of the *in vivo* studies described has the absence of AnxA1 been restricted to T cells. As such, it is not possible to attribute changes in the resultant *in vivo* response to effects of AnxA1 specifically in T cells. Studies of this nature are critical in that it is beyond doubt that the actions of AnxA1 extend well beyond T cell activation. As already described, numerous studies have demonstrated that AnxA1 acts to restrict recruitment of innate leukocyte subsets, targeting key events in the microvasculature. These leukocytes are critical “responder cells” in the development of T cell-mediated inflammation. Therefore, in addition to its effects on T cell activation, inhibition or absence of AnxA1 *in vivo* has the potential to dramatically modulate the effector response in the periphery. In addition, while early studies provided evidence of an effect of AnxA1 on regulatory T cell function (Hirata and Iwata, 1983), this concept remains to be fully explored. Similarly, unanticipated actions of AnxA1, possibly in non-immune cells, as exemplified by the observation of altered basal lung function in AnxA1^–/–^ mice (Ng et al., 2011) require further investigation. These complexities will be best addressed in studies in which AnxA1 deficiency is restricted to antigen-specific T cells, regulatory T cells, dendritic cells, or non-immune cells.

## EFFECT OF EXOGENOUS AnxA1 AND AnxA1-DERIVED PEPTIDES

Much of the interest in AnxA1 as a potential therapeutic has stemmed from results of its use as an exogenous anti-inflammatory agent in *in vivo* models of inflammation. For example, human recombinant AnxA1 has been shown to exert anti-inflammatory effects in a carrageenan-induced edema model of inflammation in the rat paw (Wu et al., 1995). However, a great deal of this work has examined the effects of peptides derived from AnxA1. From a protein of over 300 amino acids, several small peptides derived from the N-terminal region of AnxA1 retain much of its biological activity. These peptides, termed Ac2-26, Ac2-12, or Ac9-25 (constructed with an acetyl-blocked N-terminus for stability and delay of proteolytic degradation) have all been reported to retain the majority of the effects of the full-length AnxA1 protein in a number of different *in vitro* and *in vivo* systems.

The anti-inflammatory effects of peptide Ac2-26 have been demonstrated in numerous models including ischemia/reperfusion injury in both the rat (D’Amico et al., 2000; La et al., 2001) and mouse (Gavins et al., 2003), the mouse air-pouch and rat paw edema models of inflammation (Cirino et al., 1993; Perretti et al., 1993), and in models of neutrophil and monocyte trafficking (Szab’o, 1997; Bandeira-Melo et al., 2005). The wide-ranging effects of peptide Ac2-26 were clearly demonstrated in a model of pleurisy in the rat, in which the peptide inhibited mast cell degranulation, plasma protein leakage, and accumulation of both neutrophils and eosinophils (Teixeira et al., 1998). It is important to note that the evidence is consistent with peptide Ac2-26 mediating these anti-inflammatory effects by impacting on several distinct mechanisms. For example, in ischemia/reperfusion of the heart, AnxA1 modulated inflammation via effects on blood-borne cells (La et al., 2001; Gavins et al., 2005a), as well as by having direct effects on cardiomyocytes (Ritchie et al., 2003).

One of the most important techniques employed in teasing out the effects of pharmacological doses of AnxA1 and its mimetic peptides on the inflammatory cascade has been intravital microscopy (IVM). Directly imaging the microvasculature during an inflammatory response has been critical in demonstrating the ability of exogenously administered AnxA1 to reduce the capacity of leukocytes to adhere to and migrate through inflamed post-capillary venules (Lim et al., 1998; Gavins et al., 2003). Exogenous administration of AnxA1 to mice following clamping and release of the superior mesenteric artery to induce ischemia/reperfusion injury, resulted in an anti-inflammatory effect that was associated with the detachment of neutrophils from the endothelium (Gavins et al., 2003). This demonstration of an effect of AnxA1 on leukocyte recruitment is further supported by *in vitro* studies, which demonstrate that AnxA1 inhibits firm adhesion of neutrophils to human umbilical vein endothelial cells under flow conditions. However in contrast, the AnxA1-derived peptide, Ac2-26, only reduces leukocyte capture and rolling without affecting firm adhesion (Hayhoe et al., 2006) or altering increased vascular permeability (Cirino et al., 1989; Gavins et al., 2003). These findings provide evidence that the actions of peptide Ac2-26 do not entirely overlap with those of full-length AnxA1.

The examination of the therapeutic efficacy of AnxA1 has recently been extended to an OVA-induced model of antigen-induced airways inflammation (Perretti et al., 1993) This study examined the effect of a cell-permeable form of AnxA1, administered as an exogenous anti-inflammatory agent to wild-type mice during the sensitization phase of the model (Lee et al., 2012). Administration of AnxA1 conjugated to a “Tat” cell-penetrating peptide, during the latter stages of OVA sensitization, alleviated inflammation, cytokine production, airways hyper-responsiveness, and OVA-specific IgE production. Notably, while the Tat-conjugated form of AnxA1 was therapeutically effective, native AnxA1 administered in a similar fashion did not significantly reduce disease parameters. This finding suggests that the capacity of Tat-AnxA1 to enter target cells was critical in achieving its anti-inflammatory effect in this setting.

## IMPACT OF AnxA1 IN DRUG DISCOVERY

The identification of the mechanism of actions of AnxA1 (notwithstanding the unresolved question of whether the role of FPR2/ALXR is pro-inflammatory or anti-inflammatory; Perretti and Dalli, 2009), in parallel with the attractive concept of developing novel therapeutic agents based on mimicking specific endogenous pathways, has lead to an increase in drug discovery programs within this area. The ultimate aim of treatments based on AnxA1 is to retain the anti-inflammatory properties of glucocorticoids that signal for the resolution of inflammatory events, while avoiding the highly detrimental potential metabolic side effects of long-term use of exogenous glucocorticoids (Perretti and Gavins, 2003).

One strategy for this would be to identify new chemical entities from biologically-active peptide sequences from the N-terminus of AnxA1. However, from a drug discovery point of view, small molecules are a more attractive proposition due to their more attractive pharmacokinetic properties. Thus, the identification of the receptor by which AnxA1 mediates its biological effects (i.e., via FPR2/ALXR) has stimulated a great deal of interest. For example, Amgen have developed a program to identify small chemical entities that are specific for FPR2/ALXR (Perretti and Dalli, 2009). In addition, novel computer modeling approaches are now being used to identify ligands that are specific for members of the FPR family, in several cases for FPR2/ALXR (Shemesh et al., 2008; Hecht et al., 2009). The ultimate aim of this work is to identify a low molecular weight compound with the capacity to interact with FPR2/ALXR to mediate a comparable profile of anti-inflammatory effects to that of AnxA1

## CONCLUSION

Inflammatory disease affects a huge number of patients world-wide. In many cases, the therapeutic approaches in use for these patients have not changed for the last 30 years. Treatments such as exogenous glucocorticoids remain a first-line therapy for prevalent diseases such as rheumatoid arthritis. Indeed their efficacy in inhibiting inflammation ensures they remain a favored therapeutic modality, despite their well-established highly detrimental metabolic side effects. The next level of sophistication however, is to investigate the mechanisms of action of glucocorticoids in these patients, and to learn more about the endogenous pathways glucocorticoids mobilize and interact with to inhibit inflammation. AnxA1 is a prime example of this approach. The investigation of the mechanisms of action of AnxA1, as well as those of its breakdown products and receptors, holds great promise for the development of more specific novel therapies which mimic the anti-inflammatory effects of glucocorticoids, while potentially avoiding the negative effects of glucocorticoid use. AnxA1 has consistently been found to play an inhibitory role in innate forms of inappropriate inflammation. Therefore novel AnxA1-derived therapeutics are more likely to be immediately applicable in conditions such as ischemia/reperfusion injury, where the innate immune system plays a leading role. In contrast, given the previously-described inconsistency in the reported actions of AnxA1 in T cell-mediated immunity, this remains premature for forms of inflammation mediated by the adaptive immune system. However, given more detailed research in this area, AnxA1-derived therapeutics may also eventually find use in specific forms of T cell-mediated disease.

## Conflict of Interest Statement

The authors declare that the research was conducted in the absence of any commercial or financial relationships that could be construed as a potential conflict of interest.
